# Data Resource Profile: Hospital Episode Statistics Admitted Patient Care (HES APC)

**DOI:** 10.1093/ije/dyx015

**Published:** 2017-03-15

**Authors:** Annie Herbert, Linda Wijlaars, Ania Zylbersztejn, David Cromwell, Pia Hardelid

**Affiliations:** 1 Population, Policy and Practice Programme, UCL Institute of Child Health; 2 Department of Behavioural Science and Health, UCL Institute of Epidemiology and Healthcare; 3 Farr Institute of Health Informatics Research, University College London, London, UK; 4 Department of Health Services Research and Policy, London School of Hygiene & Tropical Medicine, London, UK; 5 Clinical Effectiveness Unit, Royal College of Surgeons of England, London, UK; 6 Department of Primary Care and Population Health, University College London, London, UK

## Data resource basics

### Scope

Hospital Episode Statistics Admitted Patient Care (HES APC) data are collected on all admissions to National Health Service (NHS) hospitals in England. HES APC also covers admissions to independent sector providers (private or charitable hospitals) paid for by the NHS.^1^ It is estimated that 98–99% of hospital activity in England is funded by the NHS.^2^ A hospital admission includes any secondary care-based activity that requires a hospital bed, thus including both emergency and planned admissions, day cases, births and associated deliveries. HES APC does not cover accident and emergency (A&E, emergency department) attendances or outpatient bookings; these data are held in separate HES databases. All HES databases are collated and curated by NHS Digital (previously the Health and Social Care Information Centre). In the financial year 2014/15 (April to March), 18 731 987 hospital episodes from 451 different NHS hospital trusts (known as ‘providers’) were recorded in HES APC.^3^

### Purpose of data collection

The need for national data collection on hospital activity to inform management and planning of services was first recognized in the early 1980s by a Department of Health working group.^4^ Following these recommendations, a national programme was progressively rolled out, starting in 1987 and obtaining continual national coverage by (financial year) 1989/90.^5^ Since 2004/05, HES APC has also served as the basis for ‘Payment by Results’ (PbR), a pay-for-performance system of secondary care reimbursement in the NHS internal market.^6^

### Structure

HES APC data files are structured according to financial years. Each row in HES APC indicates a ‘Finished Consultant Episode’ (FCE). An FCE represents a continuous period of care under one consultant, and each is specified with a start and an end date. Episodes are labelled as ‘finished’ and entered in HES APC according to the financial year in which they end. Consequently, episodes that start in one financial year and end in another will be classified as unfinished in the starting financial year, and finished in the ending financial year. Unfinished episodes need to be removed before analysis to prevent double counting.

A hospital admission in HES APC is referred to as a ‘spell’, defined as an uninterrupted inpatient stay at one hospital. A spell may include several FCEs if the patient was seen by multiple consultants during the same stay, but does not include transfers between hospitals. If a patient is transferred to a different hospital, a new spell begins.

In order to identify and measure continuous hospital stays, which include transfers to other hospitals, continuous inpatient spells (CIPs) need to be derived. Although CIP identifiers are not provided in standard HES APC extracts, methods for linking FCEs into CIPs are available,^7^ including that recommended by NHS Digital.^8^

### Research uses

HES APC has been frequently used for research and service evaluation, due to its universal coverage, long period of data collection and the ability to follow individuals over time. HES APC offers the opportunity to estimate population-based admission and procedure rates by condition and type of procedure, compare hospital performance and create hospital-based cohorts for short- or long-term follow-up. Since HES APC covers all births in NHS hospitals, representing 97.3% of births in England,^9^ it is also possible to create nationally representative birth cohorts.

### Processing cycle and frequency of data collection

Upon discharge from the care of a particular consultant, the treating clinician completes a discharge summary for the patient of diagnoses made and procedures carried out during that FCE (where procedures include surgery, diagnostic imaging, ventilation and infusion/transfusion therapy). Discharge summaries are forwarded to a clinical coding department in the hospital, who enter the information onto the local electronic patient information database. Clinical coders undergo nationally accredited training programmes and follow standardized rules for translating information on discharge summaries into clinical codes.^10^^,^^11^

Every month, data are extracted from local hospital databases to the Secondary User Service (SUS), a national data warehouse housed within NHS Digital.^12^ Data from the SUS are extracted both for purposes of hospital reimbursement under PbR, and separately to create a provisional monthly HES extract. NHS Digital carry out basic data checks and cleaning, add geographical fields based on patient postcodes, and attach pseudonymized patient identifiers (‘HESIDs’) to each episode.^13^^,^^14^ At the end of each financial year, NHS Digital allow hospitals one further data submission to HES (the ‘Annual Refresh’), after which a provisional annual HES extract is produced for final review by hospitals. Once the Annual Refresh has been checked, a final annual HES dataset is made available.^12^

### Linkage within HES APC

From 1997/98 onwards (when patients’ NHS numbers became a mandated return from hospitals), HES APC episodes have been linked longitudinally to the same patient by tagging episodes with the HESID. This alphanumeric variable allows patient follow-up, yet avoids the need for supplying patient identifiers to researchers. The methods used to generate the HESID have been described elsewhere.^15^ Each HES APC extract contains a unique set of HESIDs to reduce the risk of individual disclosure through merging separate data extracts supplied to different research teams.

### Linkage to other datasets

HES APC data can be linked to other datasets held by NHS Digital, including HES A&E attendances (from 2007/08), HES Outpatient appointments (from 2003/04), adult critical care (from 2008/09), diagnostic imaging data (covering all radiology procedures from 2012/13), the Mental Health Services Dataset (for all adult community and outpatient mental health care contacts from 2006/07) and Patient Reported Outcome Measures (pre- and postoperative questionnaires filled out by patients undergoing knee or hip replacements, varicose vein surgery or groin hernia repair from 2009/10). Secondary users can link these datasets because the same HESID algorithm is applied to each dataset.

HES APC is also routinely linked to a number of external datasets. The Clinical Practice Research Datalink,^16^ a large UK primary care database, is linked to HES APC on a monthly basis. HES APC is linked to dates and causes of non-hospital deaths from the Register of Deaths in England and Wales held by the Office for National Statistics (for deaths registered since 1 January 1998), also on a monthly basis.^17^ Only deaths of patients recorded in HES APC are available through this linkage (i.e. deaths of persons who have not had a hospital admission since April 1997 are not included).

NHS Digital also provides a trusted third-party bespoke linkage service, through which secondary users can request that HES APC data be linked to other external datasets. For example, both national disease registries (such as the National Joint Registry^18^ and the UK Renal Registry^19^) and well-established cohort studies including Whitehall II^20^ and the Hertfordshire Cohort Study^21^ have been linked to HES APC. Secondary users need to obtain the appropriate approvals to enable these linkages.

## Measures

### Clinical and patient data

HES APC provides detailed clinical, demographic and organizational information for each FCE (see Table 1), with 270 variables available in the core dataset. Apart from data on diagnoses and procedures, HES APC contains information on dates of admission, operations and discharge, admission method (e..g. emergency or planned), care provider and many geographical variables mapped from a patient’s postcode. The local health geographies and hospital providers in England have changed several times since 1997, and thus care needs to be taken to ensure continuity when carrying out local or provider level analyses that use HES APC data covering many years.

**Table 1:** Selection of key data fields available for each finished consultant episode (FCE) in HES APC data^22^

Patient	Admission/FCE	Clinical	Geography	Provider/ organisational	Maternity/birth (only in maternity tail)
HESID Age at admission Age at discharge Sex Ethnic group	Episode start date Episode end date Date of admission Date of discharge Admission method (e.g. - planned, emergency, birth) Discharge method Admission source Discharge destination Waiting time (from date of decision to admit to date of admission)	Diagnoses (up to 20) Operations (up to 24) Operation dates (up to 24) Consultant specialty (admitting and treating consultant)	Government office region Local authority Clinical commissioning group Index of multiple deprivation (IMD) 2004 rank, deciles and domains	Care provider (hospital) General practice of patient	Gestational age Number of previous births Birth weight Maternal age Mode of delivery Baby number (for multiple births)

Socioeconomic status is measured by the Index of Multiple Deprivation 2004 (IMD), a small area-based indicator constructed from several different measures of deprivation.^22^ IMD is measured at Lower Super Output Area (LSOA) level, where an LSOA contains between 400 and 1200 households.^23^ Individual-level measures of socioeconomic status (e.g. education level or income) are not available. Detailed information on variables available, specific cleaning rules and coding used are available in the HES APC Data Dictionary provided by NHS Digital.^24^

Diagnoses are coded using the International Classification of Diseases version 10 (ICD-10).^25^ ICD-9 was used between April 1989 and March 1995. The number of diagnosis fields has increased over time: since April 2007, each FCE can have up to 20 ICD-10 codes entered (up from 7 codes before April 2002 and 14 in April 2002–March 2007). Each FCE has one primary diagnosis, which accounts for the majority of the length of stay of the FCE. The other diagnoses are referred to as comorbidities. According to NHS Digital cleaning rules, each FCE must have at least one primary diagnosis, although it may be recorded as unknown (ICD-10 code R69).

Operations and other interventions are coded using a UK-specific system, the Office of Population Censuses and Surveys Classification of Interventions and Procedures (OPCS, currently version 4.7).^26^ This has evolved over time as new techniques and technologies have been introduced. A history of versions in use is available from the NHS Digital coding standards website.^26^ Each FCE may have up to 24 operations recorded (up from 4 before April 2002 and 12 in April 2002–March 2007), but procedure fields are left empty if patient management did not require an intervention covered by OPCS (e.g. where the primary treatment was a drug regimen or observation). A primary procedure is selected for each FCE as that which is the most resource-intensive, but a procedure may be described using more than one code to indicate surgical approach, anatomical location and side of procedure (e.g. stent placed under radiological control in femoral artery of left leg). Dates are also entered for each procedure.

### Birth and delivery information

Each birth event in HES APC generates at least two FCEs: one delivery episode and one or more birth episodes. Each delivery and birth episode includes an additional ‘maternity tail’, with detailed fields including the baby’s birthweight, gestational age, birth order (for multiple births), mode of delivery and maternal age (Table 1). The maternity tail is based on information entered via local maternity databases. Unlike the diagnostic and procedure fields, the maternity tail data fields use HES-specific categories rather than standardized classifications, and it is not a mandated return to NHS Digital. This leads to large variations in data completeness and quality.^27^^,^^28^ It is not possible to directly link a mother and a baby in HES APC; that is, the mother’s HESID is not copied to the baby’s birth record. However, linkage between mother and baby is possible using probabilistic methods.^29^

### Hospital use in England

**Figure 1:**
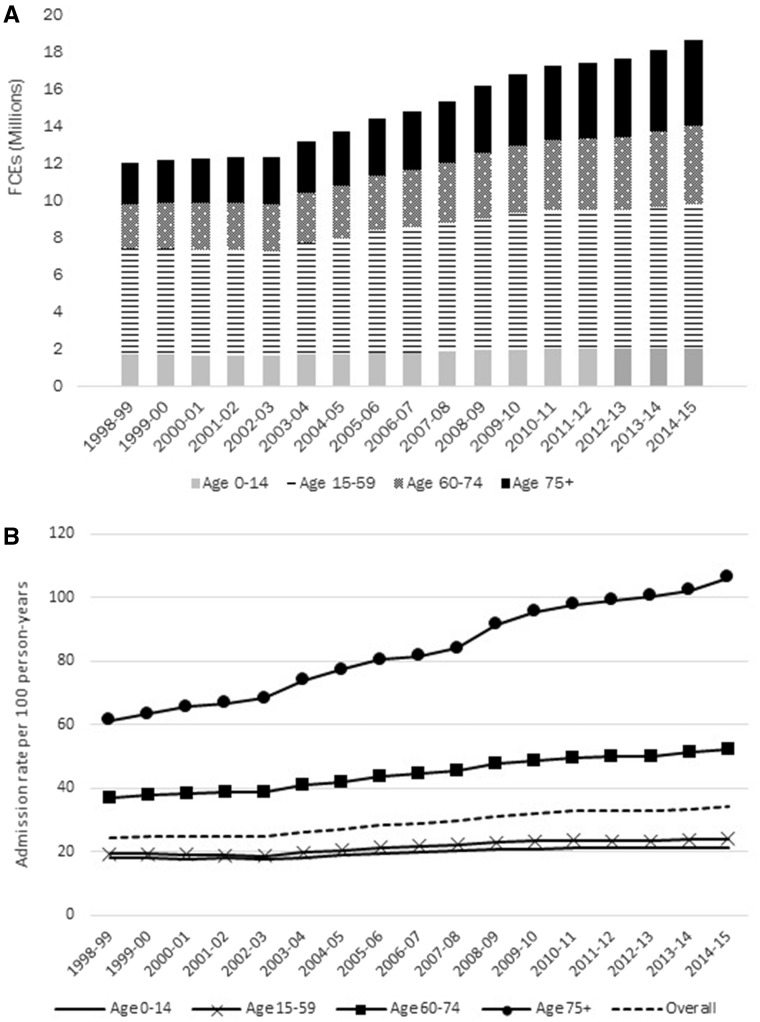
A) Number of finished consultant episodes (FCEs) by age group from financial years 1998/99 to 2014/15; and B) episode rates by age group per 100 person-years. Denominators for rates are based on mid-year population estimates for England^78^.

Both numbers and rates of hospital admissions have increased during the period of HES APC data collection (Figure 1), particularly among older adults (aged 60-74 and 75+). Between 1998/99 and 2014/15, the overall FCE rate has increased by 40% from 24.5 per 100 person-years to 34.3 per 100 person-years, with the steepest increase (73.0%) in adults aged 75+.

Since HES APC covers all hospital admissions, infants and older adults (aged 65+) are over-represented in HES APC compared with the general population of England (Table 2).

**Table 2:** Demographic characteristics of HES APC patients compared with general population of England

Characteristic	HES APC^a^	England^b^
Finished consultant episodes	18731964	
Admissions	15892434	
Admission type		
Emergency	5615707 (30.0)	
Waiting list	6119234 (32.7)	
Planned	2154564 (11.5)	
Other	2002929 (10.7)	
Sex		
Male	8359362 (44.6)	26773200 (49.3)
Female	10370245 (55.4)	27543400 (50.7)
Gender unknown	2357 (0.01)	–
Age		
0 years	1013476 (5.4)	664183 (1.2)
1–4 years	454461 (2.4)	2766774 (5.1)
5–14 years	568902 (3.0)	6245420 (11.5)
15–24 years	1167439 (6.2)	6837371 (12.6)
25–34 years	1880715 (10.0)	7425591 (13.7)
35–44 years	1573273 (8.4)	7103408 (13.1)
45–54 years	1986116 (10.6)	7635651 (14.1)
55–64 years	2319214 (12.4)	6100512 (11.2)
65–74 years	3013044 (16.1)	5162873 (9.5)
75–84 years	2941250 (15.7)	3099319 (5.7)
85+ years	1711354 (9.1)	1275516 (2.3)
Missing	102720 (0.5)	

Numbers within parentheses represent proportions of FCEs (for HES APC) and proportions of persons (for England)

^a^Data source: HES APC 2014–15.^3^

^b^ONS 2014 mid-year population estimates.^75^

## Data resource use

Although no up-to-date bibliography of published research based on HES APC is curated by the data providers, a 2013 systematic review identified 148 articles using HES APC data published between 1989 and July 2011.^30^ We carried out a subsequent search on PubMed on the 8 June 2016 using the search term ‘Hospital Episode Statistics’ for article abstracts published since July 2011. We identified 264 relevant publications where the primary analysis involved the use of HES APC data, and a further 130 papers where HES data had been linked to cohorts created in other datasets. The annual number of publications using HES APC data has increased from 2 in 1993^30^ to 88 in 2015.

Published studies using HES APC data have covered a diverse range of topics. They have explored the incidence of conditions across regions and over time.^31^^,^^32^ They have also examined cross-sectional and longitudinal patterns of treatment by organization,^33^ including comparing NHS and privately contracted providers^34^ or regions,^35^^,^^36^ both from descriptive and analytical perspectives. Regional comparisons have included evaluating the impact of clinical evidence^37^ or guidelines^38^ as well as health care policies.^39^ They have examined the outcome of medical as well as surgical therapies (such as survival,^40^ short-term postoperative mortality,^41^ complications,^42^ reoperation^43^ and hospital readmissions^44^), with some seeking to identify factors that are associated with these outcomes, in terms of both patient characteristics^45^^,^^46^ and organizational factors such as surgical volume^47^ or day of week.^48^ Methodological studies include creating coding frameworks,^28^ applying comorbidity scores,^49^ developing risk prediction models^50^ and using look-back methods to impute missing data items.^51^

Many high profile routinely produced reports on the quality of secondary care are based on HES APC data. These include hospital mortality monitoring reports produced by NHS Digital^52^ and commercial organizations,^53^ and research reports by independent think-tanks^54^ and Royal Medical Colleges.^55^

## Strengths and weaknesses

### Coverage

The key strength of the HES APC database is its universal coverage, which provides an unselected sample of hospital episodes. The large size of HES APC makes it possible to precisely estimate admission rates and capture outcomes for rare conditions, including congenital anomalies or specific cancers.

### Longitudinal linkage

Another strength is the possibility to longitudinally link patients using the HESID, allowing for the creation of HES-based cohort studies if a suitable inception date can be identified. The long period of data collection of HES (currently up to 19 years) allows long-term follow-up of admitted patients, which has allowed the development of risk prediction models for distal outcomes.^44^

### Standardized coding

ICD-10 coding of clinical diagnoses offers the opportunity to use HES APC for international comparisons of secondary care use. Since ICD-10 is used in hospital administrative data across the UK, Europe, Canada, Australia and New Zealand, HES APC has been used to assess the impact of differential health policy between NHS systems and internationally.^56–58^ International studies using HES APC include cross-country comparisons of the incidence of neonatal abstinence syndrome^59^ and non-small cell lung cancer.^60^ Nonetheless, international comparisons are challenging due to differences between countries in admission thresholds, organization of care provision, and whether secondary care is free at point of use or requires health insurance or other payment.

HES APC episodes are readily linked to information on costs of care, due to the ability to match each episode to a Healthcare Resource Group, and hence a unit cost.^61^ This makes HES APC an important data resource for health economics.^62–64^

### Coding variation

One of the key challenges in interpreting HES APC is the reliance on diagnostic and procedure codes for identifying study participants and outcomes. Despite centrally issued coding rules, clinical coders rely on the quality and detail of completed discharge summaries to enter data consistently. Consequently, diagnostic coding practices vary between hospitals, particularly for comorbidities.^65^

Since the roll-out of PbR, financial incentives now exist for hospitals to improve coding depth in order to ensure accurate reimbursement. This has led to an increase in the number of diagnostic codes used and improvements in coding accuracy.^7^^,^^66^ The introduction of PbR therefore poses challenges for interpreting time-series studies using HES APC data, and care must be taken to not overinterpret results identifying increasing complexity of cases admitted.^7^

### Sensitivity to admission thresholds

Since HES APC covers only admitted patients, it is sensitive to variation between hospitals or over time in admission thresholds. The introduction of the four-hour waiting target in A&E departments in 2004 has been suggested as a contributing factor for the increase in rates of emergency admissions in children during the 2000s.^67^^,^^68^ Changes in thresholds for emergency admissions can be examined using linked HES A&E data;^69^ however, variation in admission thresholds for planned procedures cannot readily be determined using HES datasets.

### Missing data

Although age, sex and clinical characteristics are well completed in HES APC (see Table 2), data on ethnicity are not. Ethnicity has been a mandated return for all NHS contacts since 1991. Although ethnicity recording has improved over time, the proportion of patients with a known ethnicity recorded was still only 85% in 2011, up from 41% in 1997.^70^

**Figure 2:**
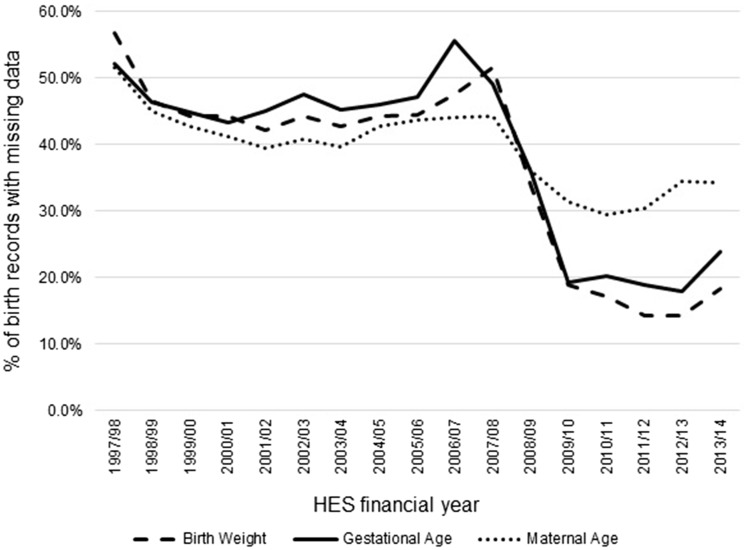
Proportion of birth records with missing data for selected variables in the maternity tail from financial years 1997/98 to 2013/14.

Further, there is a high proportion of missing data in the maternity tail fields (see Figure 2). Postcodes were not extracted from the SUS for birth episodes prior to 2013/14, which means earlier birth episodes cannot be mapped to geographical variables, including the Index of Multiple Deprivation (IMD).^71^ As an example, completeness of the IMD decile variable for singleton birth episodes in 2012/13 was 7.8%, compared with 81.9% in 2013/14.

### Quality of internal linkage

The HESID linkage algorithm relies heavily on the accurate recording of NHS number across all hospital episodes to avoid missed matches (FCEs that have failed to link to a patient). Consequently, there is a substantial proportion of missed matches in HES APC. A recent estimate puts the HESID missed-match rate at 4%,^72^ leading to an underestimation of readmission rates by 3.8%. NHS numbers were not provided at birth until 2002, meaning that linkage within HES APC and to other HES and external datasets is not reliable for births before 2002/3.^73^

### Scope limitations

HES APC covers higher dependency (HDU) or intensive care unit (ICU) periods, but it does not contain ‘flags’ to identify such stays, nor detailed information on level of care or HDU/ICU interventions. A separate HES dataset covers adult critical care from 2008/09,^74^ whereas data relating to neonatal or paediatric intensive care are collected through systems external to NHS Digital.

Data on drugs prescribed through hospital pharmacies to inpatients are not available in HES APC. There is currently no national individual-level hospital prescribing database for England.

### Opt-outs

Patients who do not wish their records to leave NHS Digital can lodge a ‘type 2 opt-out’ with their primary care practice.^75^ From 29 April 2016, any records (including in previous financial years) relating to persons who have opted out in any NHS Digital dataset (including HES APC) will therefore be removed before supply to secondary users. Overall, for the 2014/15 HES APC annual extract, 2.3% of episodes will be removed, with substantial geographical variation in opt-out rates.^75^

## Data resource access

Access to HES APC data is provided by NHS Digital for the NHS, government, researchers and commercial health care bodies. Those requesting an extract of the data must show that their work will support health and social care and improve health.^76^ Data cannot be released for solely commercial purposes.

Data are requested through the online Data Access Request Service (DARS). Applications are evaluated by the Data Access Advisory Group which check all data requests for patient-level data to evaluate whether there is an appropriate legal basis for data dissemination and that appropriate data security is in place. Details about HES applications and associated costs are available on the DARS website [http://content.digital.nhs.uk/DARS].

NHS Digital carries out audits to check that data users meet obligations regarding the terms and conditions of use, including disclosure control.^77^

**Profile in a nutshell**

- HES APC contains data on all admissions to National Health Service (NHS) hospitals in England, or to independent hospitals where the costs are met by the NHS. It was originally set up for purposes of management and planning of hospital services. Data are now also collected for purposes of reimbursing hospital activity.
- HES APC includes all hospital care episodes from the financial year 1989/90 onwards (1 April 1989–31 March 1990). Pseudonymized patient identifiers that allow for longitudinal follow-up of patients are available from 1997/98 onwards.
- HES APC data are entered from medical records by clinical coders in each hospital, according to national clinical coding standards. The database is collated and processed centrally by NHS Digital (previously the Health and Social Care Information Centre).
- Data fields exist for diagnoses, procedures, patient demographics (including ethnicity and area-level deprivation), admission and discharge dates, hospital and other variables.
- HES APC data can be linked to outpatient and emergency department attendances as well as datasets external to NHS Digital, including death registrations.
- Aggregate data are accessible via the NHS Digital website and individual-level data are available through the NHS Digital Data Access Request Service, subject to approval and a cost recovery charge.

## Funding

L.W. is supported by funding from the Department of Health Policy Research Programme through funding to the Policy Research Unit in the Health of Children, Young People and Families (grant reference number 109/0001). This is an independent report commissioned and funded by the Department of Health. The views expressed are not necessarily those of the Department. A.Z.’s PhD studentship is supported by awards to establish the Farr Institute of Health Informatics Research, London, from the Medical Research Council, Arthritis Research UK, British Heart Foundation, Cancer Research UK, Chief Scientist’s Office, Economic and Social Research Council, Engineering and Physical Sciences Research Council, National Institute for Health Research, National Institute for Social Care and Health Research and Wellcome Trust (grant MR/K006584/1). P.H. is funded by a National Institute for Health Research postdoctoral fellowship (number PDF-2013‐06‐004). This article represents independent research funded by the National Institute for Health Research (NIHR). The views expressed are those of the authors and not those of the NHS, the NIHR or the Department of Health.

**Conflict of interest:** None declared.
